# Climate-friendly food-choice intentions among emerging adults: extending the theory of planned behavior with objective ambivalence, climate-change worry and optimism

**DOI:** 10.3389/fpsyg.2023.1178449

**Published:** 2023-06-20

**Authors:** Kirsti M. Jylhä, Maria Ojala, Sandy Odisho, Anja Riise

**Affiliations:** ^1^Institute for Futures Studies, Stockholm, Sweden; ^2^School of Behavioural, Social and Legal Sciences (Psychology), Örebro University, Örebro, Sweden

**Keywords:** young people, pro-environmental behavior, food choices, climate-change worry, attitudinal ambivalence

## Abstract

Climate-friendly food choices are still relatively rarely addressed in studies investigating climate engagement, particularly among young people. To address this research gap, we conducted a questionnaire study with senior high school students (*N* = 474). Our overarching theoretical framework is the Theory of Planned Behavior (TPB), which we extended with emotional factors (climate-change worry and optimism) and attitudinal ambivalence. We found that all factors included, except for optimism, correlated with the food-choice intentions. In multiple regression analyses, worry was the second strongest predictor, after attitudes. Moreover, a measure of objective ambivalence moderated the correlation between attitudes and intentions by weakening it. The results support the validity of using the TPB model when explaining intentions to make climate-friendly food choices among emerging adults. However, our results suggest that it is also important to consider emotions—in this case climate-change worry—and the existence of conflicting evaluations about choosing climate-friendly food.

## 1. Introduction

There is widespread interest and worry regarding climate change among young people (Hickman et al., 2021; Léger-Goodes et al., 2022). This is not surprising considering that they will face the increasingly worsening consequences of climate change during their lifetimes. Moreover, young people are relatively knowledgeable about certain aspects about climate change, because the topic is included in the school curriculum in many countries (Ratinen, 2021). However, not all young people are concerned about the issue, and even if they are, they may still contribute to worsening climate change through their lifestyle choices and consumption patterns. For example, it is not always easy for young people to make climate-friendly food choices even if they are worried about climate change because our society is characterized to a large extent by unsustainable social norms and habits (see Ojala, 2022). Indeed, although much of the focus of public discussions has been on acknowledging the distress and public demonstrations (e.g., Fridays for Future) among young people, this age group is not homogeneous in terms of responses to climate change, and in general they do not tend to have more sustainable lifestyles than older populations (Grønhøj and Thøgersen, 2009; Hyry, 2021). It is thus vital to understand what factors can hinder or promote climate engagement in this group.

A person can help mitigate climate change in their everyday life in many ways, one of which is to make climate-friendly food choices. This is one of the most effective forms of individual climate action (Wynes and Nicholas, 2017; Poore and Nemecek, 2018) but is still relatively rarely addressed in studies investigating climate engagement, particularly among young people (for an exception see Mäkiniemi and Vainio, 2013). The aim of this paper is to examine some possible factors that could influence intentions to make climate-friendly food choices among young people in late adolescence. We focus on the factors included in the Theory of Planned Behavior (TPB) and extend it with emotions regarding climate change (worry and optimism) as well as ambivalent attitudes. Late adolescence, also called emerging adulthood, is a particularly interesting age period when considering climate engagement, because it is a transitional phase where values, norms, and identities are not yet established, and old habits can be challenged and new ones can emerge (Verplanken et al., 2018). It is not certain that the same factors that are important for older adults are the most vital for late adolescents and young adults when it comes to motivating climate-change action. This could be due to both developmental factors and the specific time-period when a person is in the formative years of adolescence (Pereira and Freire, 2021).

## 2. Theoretical and empirical background

### 2.1. Theory of planned behavior

Our overarching theoretical framework is the Theory of Planned Behavior (TPB; Ajzen, 1991). TPB is one of the most widely used frameworks to explain behavioral outcomes and intentions (Armitage and Conner, 2001), including environmentally significant ones, such as sustainable consumption, green hotel choices, energy and water saving, forest conservation, and recycling (Kollmuss and Agyeman, 2002; Vermeir and Verbeke, 2008; Han et al., 2010; Turaga et al., 2010; de Leeuw et al., 2015; Yuriev et al., 2020; Savari and Khaleghi, 2023). In the present study, we focus on the intention to perform a behavior which, according to the TPB model, is the most proximal predictor of the actual behavior. Three antecedents are expected to predict intention (and indirectly predict behavior): attitudes, subjective norms, and perceived behavioral control (Ajzen, 1991). Attitudes toward a behavior develop from beliefs an individual holds about the behavior in question. These beliefs concern different evaluations of the behavior (if it is desirable or not, something that one values or not, and so on) and together form either a positive, negative or neutral attitude toward the behavior. Subjective norms are formed on the basis of perception of how one’s significant others expect one to behave. Perceived behavioral control relates to the individual’s conviction of their own capability to perform the behavior in question.

The TPB model considers the complexity of behavioral decisions, meaning that positive attitudes regarding the outcomes of the behavior may motivate behaviors, but individuals also have a need to feel that they are able to perform a behavior and that others who they care about approve and encourage it. Similar reasoning can be extended to food choices, which is a type of behavior that is regulated by strong personal habits, cultural traditions, and social norms (Macdiarmid et al., 2016; Carrus et al., 2018; Salmivaara et al., 2022). Indeed, the TPB model explains health-related dietary choices and behavioral intentions among adolescents (Grønhøj et al., 2013; Riebl et al., 2015; Chan et al., 2016). Moreover, the few studies that have focused on environmentally motivated food choices found that the model predicts reducing meat consumption and eating locally and organically produced foods among adults among adults and young people (Bissonnette and Contento, 2001; Yadav and Pathak, 2016; Çoker and van der Linden, 2020). However, it is currently unclear if the TPB is a predictive model for adolescents’ climate-friendly food intentions as, to the best of our knowledge, no research has yet been conducted on this matter. Moreover, importantly, there is still some capacity to extend the model. As highlighted by Kollmuss and Agyeman (2002), reasons to engage in pro-environmental behaviors are so complex that they cannot be comprehensively captured by a single framework.

In this study we investigate if the basic TPB model can be extended with some factors that are of importance in the climate context. First, people are more prone to engage in climate action if they have positive outcome expectancy, meaning that they think their actions help mitigate climate change (Macdiarmid et al., 2016). For instance, a study by Roberts (1996) found that the strongest predictor of ecologically conscious consumer behavior was a belief that consumers, as individuals, can help solve environmental problems. In climate discussions, the effectiveness of individual behaviors in climate-change mitigation is commonly debated and questioned (Brownstein et al., 2022). There are also knowledge gaps regarding the actual effects of different forms of climate action among the public (Wynes and Nicholas, 2017). For example, a study by Wynes et al. (2020) found that almost half of the participants incorrectly estimated that vegan diet only has a low impact on climate-change mitigation (see also Macdiarmid et al., 2016). Thus, we measure behavioral control with outcome expectancy rather than self-efficacy in our study.

Second, we note that the basic model of the TPB views human nature as highly rational in a very specific way, focusing on conscious evaluations of costs, benefits, and own capacities in engaging with a particular behavior (see also, e.g., Kollmuss and Agyeman, 2002; Koenig-Lewis et al., 2014). The model, for example, does not consider the influence of emotions or the existence of attitudinal ambivalence. We thus extend the model by including these factors, which are central in explaining environmental outcomes, as will be explained in the next sections.

### 2.2. Climate-change worry and optimism

Emotions are reactions to things that happen in the external (or internal) environment; they reveal if a situation is in line with important values or instead a threat toward these and they are strong motivational forces (Frijda, 1986). Emotions of different kinds have also been found to be related to pro-environmental behavior (see Brosch, 2021; Brosch and Steg, 2021; Wong-Parodi and Feygina, 2021; Pihkala, 2022; Ojala, 2023). With regards to climate change the sense of climate threat can activate difficult feelings such as worry and anxiety, but people can also feel hopeful and optimistic regarding humanity’s ability to resolve the situation and to succeed in climate-change mitigation (Ojala, 2008; Pihkala, 2022). It seems possible that if people are emotionally engaged with the climate issue, they are motivated to change their environmentally detrimental lifestyle habits and that this influence goes beyond social norms and outcome expectancy. Thus, emotions could have a unique effect on the intention to make climate-friendly food choices even when accounting for the effects of other variables in the TPB model.

Regarding worry and anxiety, the theory of affective intelligence claims that these emotions, as parts of ancient defense systems, are the ones that make people rational through becoming motivated to deliberate about breaking behavioral patterns that are often habitual (Marcus et al., 2000). This theory has mostly been used in political science to explain involvement in different kinds of societal issues. It could be particularly important for understanding the potentially constructive role of the emotions of worry and anxiety also regarding climate-change engagement, due to its focus on these emotions as highly rational reactions (see Ojala et al., 2021). Indeed, there are rather consistent results showing a positive association between climate-related worry and pro-environmental behavior (Bouman et al., 2020; Ogunbode et al., 2022; Simon et al., 2022). However, what the relationship looks like concerning climate-friendly food choices among young people has not been investigated before.

As to hope and optimism, their role in explaining climate engagement is unclear (see, e.g., Ojala, 2023). Both optimism as a personality trait (Kaida and Kaida, 2019) and general state optimism (MacKinnon et al., 2022) have been found to be positively related to pro-environmental behavior among adults. Regarding climate-related hope and optimism, that is, a view that humanity can fight climate change in a successful way, the correlation with climate engagement has been positive in some studies (Feldman and Hart, 2018; Pihkala et al., 2022; Sangervo et al., 2022), but non-significant in some other studies (Hornsey and Fielding, 2016; Wang and Chen, 2022). Moreover, in one study an optimistic message was less successful than a pessimistic message in increasing motivation to mitigate climate change (Hornsey and Fielding, 2016). One possible explanation for these inconsistent results is that these correlations could be difficult to estimate without simultaneously considering the presence of worry (Ojala, 2008). If optimism is experienced without experiencing worry, this may relate to a low sense of urgency and thereby less engagement in climate action. Ojala (2008) found that hope about global environmental problems, a concept related to optimism although not exactly the same (see Snyder et al., 2002), interacted with worry to predict recycling. In the present study, we thus examine the main effects of climate-related worry and optimism, as well as of their interaction, on the intention to make climate-friendly food choices.

### 2.3. Attitudinal ambivalence

Another critical shortcoming in the rationality assumptions of the TPB model is that it does not take into consideration that people may simultaneously have both positive and negative views regarding a specific behavior. Both climate change and the actions people can take to mitigate it are very complex and thus can evoke ambivalent attitudes, that is, mixed positive and negative views and emotions about the specific behavior (Conner and Sparks, 2002; Ojala, 2008). This is particularly salient in the context of food choices: The meaning of food is only partly based on its nutritional value, and diet changes can be seen as inconvenient, and even a threat to hedonistic pleasures, culinary traditions, and certain identities (Povey et al., 2001; Sparks et al., 2001; Berndsen and Van der Pligt, 2004; Rosenfeld and Tomiyama, 2021). Thus, when asked to consider the climate crisis when making food choices, people can experience many kinds of conflicts. These conflicts can be related to, for example, clashes between environmental values and self-interest (e.g., enjoyment of the taste of meat) and feelings of helplessness and a lacking sense of efficacy in the face of a crisis of this scale (Macdiarmid et al., 2016; Ojala and Anniko, 2020; Ojala, 2022). Food choices have only relatively recently been broadly discussed in the climate context (Wynes and Nicholas, 2017), which means that people are still only starting to explore this lifestyle choice as a potential way of taking personal climate action. Indeed, Macdiarmid et al. (2016) found that there is more resistance to diet change than to other possible climate actions.

Ambivalent attitudes and feelings have been found to be disincentives to climate-friendly food choices among young adults (Ojala and Anniko, 2020). Whether this is also the case among late adolescents has not been investigated before, however. In addition, in Ojala and Anniko’s study, only a simple measure of subjective ambivalence was used. Thus, the fact that people are not always consciously aware of their ambivalent attitudes was not considered. In the present study we acknowledge this and instead use a measure of objective ambivalence (Conner and Sparks, 2002). We assess the positive and the negative dimensions of the attitude separately – including both cognitive and affective aspects – and thereafter construct an index to capture mixed evaluations (Thompson et al., 1995). We investigate whether this objective ambivalence has a direct negative relation to climate-friendly food choices. Furthermore, research conducted on dietary choices has identified objective ambivalence as a potential moderator of the correlations between attitudes and self-reported eating of, and/or the intention to eat, vegetarian food (Povey et al., 2001) or healthy food (Conner et al., 2003), or to consume less chocolate and meat (Sparks et al., 2001). More specifically, ambivalence was found to weaken the relationship between attitudes and intention. There could also be an interaction between affective states and attitudinal ambivalence. For example, Wang et al. (2020) found that the negative correlation between ambivalence and green purchase intention was weakened by anxious mood. The authors suggested that when individuals experience anxious mood, they aim at reducing this state by taking action and that this effect is more pronounced in the presence of more stable attitude–behavior consistency, that is, lower ambivalence. We therefore also investigate whether objective ambivalence moderates the effects of attitudes and climate worry on intentions to choose climate-friendly food.

## 3. Aim, research questions, and hypotheses

The aim of this paper is to examine what factors predict the intention among late adolescents to make climate-friendly food choices. As our first two research questions (R1 and R2), we test whether the variables in the basic TPB model (attitudes, subjective norms, and in this case, outcome expectancy) (R1) and the extended model (objective ambivalence and worry and optimism regarding climate change) (R2) correlate with intentions to make climate-friendly food choices. Furthermore, we investigate whether all these variables have unique significant effects on the food-choice intentions when controlling for each other in a regression analysis (R3) and, if so, which of them is the most important unique predictor (R4). Finally, we examine whether some of the variables included interact in predicting food-choice intentions (R5). More specifically, we test whether climate-related worry interacts with climate-related optimism, and whether objective ambivalence interacts with attitudes and with climate worry. Across the analyses, we include gender as a control variable because previous research has consistently found that women tend to express more environmental concern, climate engagement, and openness to plant-based diets than men (e.g., Kollmuss and Agyeman, 2002; Rosenfeld and Tomiyama, 2021).

Based on earlier research, reviewed above, we expect that all the factors of the basic TPB model (attitudes, subjective norms, and outcome expectancy) are significantly positively related to food-choice intentions [Hypothesis 1 (H1)]. As to the variables in the extended model, we expect that objective ambivalence is significantly negatively related, and climate-change worry significantly positively related, with the intention to make climate-friendly food choices (H2). We do not form a hypothesis regarding optimism, as earlier research shows mixed results, and because it can be expected to interact with worry (see below). As to the unique effects of the predictor variables, we expect that the variables of the basic model have unique effects on the intention to make climate-friendly food choices (H3). We also expect that climate-related worry has a unique effect on food choice even when controlling for the factors of the basic TPB model (H4). However, we did not form a hypothesis regarding the unique effect of objective ambivalence because previous research has shown inconsistent results (Sparks et al., 2001), and because this variable was included in the analyses to examine possible moderation effects (see below). We also offer no hypothesis about which factors have the strongest effect on food-choice intention when controlling for the other variables. As to the interaction effects, we test three hypotheses: First, that worry moderates the effect of optimism, meaning that when people experience a high degree of worry at the same time as they are highly optimistic, they express a stronger intention to choose climate-friendly food than if they are high on optimism but low on worry (H5); Second, that objective ambivalence moderates the effects of climate worry (H6) and attitudes (H7), meaning that when people simultaneously hold positive and negative views about making climate-friendly food choices, the positive effect of their climate worry and their attitude on the food-choice intentions are weaker.

## 4. Methods

### 4.1. Participants and procedure

Participants (*N* = 474) were students in upper-secondary school from cities and towns of varying sizes in central parts of Sweden. The mean age of the participants was 17.9 (SD = 0.68), and 58.4 percent reported themselves as female and 40.7 percent as male. Only very few participants (0.8%) reported their gender as ‘other,’ and this response was therefore handled as missing value. An additional 8 persons participated in the study but were excluded from the analyses either due to unreliable response patterns or because their age was outside the age group in focus. The response rate of the study was 72.9%, as 661 students were expected to participate.

Recruitment of participants was conducted by initially contacting the principals of several upper-secondary schools. The principals received information regarding the aim and procedure of the project as well as enclosed forms regarding informed consent. The forms were asked to be uploaded to the students’ electronic platforms. Participants filled out the questionnaire during mandatory class time. The allocated time to fill out the questionnaire was 2 × 45 min with a break in the middle, during which the students received snacks and a beverage. Regardless of the break, we must acknowledge that the long time it took to answer the questionnaire could have influenced the answers to the questions in the end of the questionnaire for some of the students (Gibson and Bowling, 2020). All the items in this study were, however, presented in the first half of the questionnaire. Students who decided not to partake in the survey were encouraged to do other schoolwork during the same time. Before the survey was handed out, via a link, respondents were informed by trained test leaders about the purpose of the survey. Forms regarding informed consent were handed out to the students. They were asked to carefully read the forms and if they began to answer the survey their consent had been given. The test leaders remained present the entire time. The electronic survey was provided by Örebro University’s software Oru-Survey. The students filled out the survey on their own school computers or cell phones. For confidentiality reasons, teachers were not present during data collection. The study was approved by the Swedish National Ethics Committee.

### 4.2. Measures

The used items and scales are presented in the Supplementary material. Our dependent variable was *intention* to make climate-friendly food choices (two items, *α* = 0.87, *M* = 4.15, SD = 1.80, scale range = 1–7, Mäkiniemi and Vainio, 2013). Predictor variables in the original TPB model were *attitudes* to climate-friendly food choice (two items, *α* = 0.81, adapted from Sparks et al. (2001), *M* = 3.43, SD = 0.84, range = 1–5) and *subjective norms* (three items, α = 0.75, adapted from Pedersen et al. (2015), *M* = 2.51, SD = 0.96, range = 1–5). As to the variables that modify the model, we included a measure for *outcome expectancy* (two items, *α* = 0.81, adapted from Mead et al. (2012), *M* = 3.24, SD = 0.92, range = 1–5), *climate-change worry* (five items, *α* = 0.89, Ojala, 2012, *M* = 4.17, SD = 1.24, range = 1–6) *optimism* (three items, *α* = 0.84, Ojala, 2012, *M* = 3.20, SD = 1.02, range = 1–6), and *objective ambivalence* (Conner and Sparks, 2002; Ojala, 2008, *M* = −0.65, SD = 1.88, range = −5–5) which was calculated on the bases of positive (five items, *α* = 0.91) and negative views (five items, *α* = 0.89), using the Griffin formula (Thompson et al., 1995).^1^

## 5. Results

### 5.1. Correlations between the variables

Answering our first research question (R1), we examined zero-order correlations. As illustrated in Table 1, all the variables of the basic TPB model—that is, attitudes, subjective norms, and outcome expectancy—were significantly positively related to intention to make climate-friendly food choices. Thus, the more positive attitudes the young people have about the importance of making climate-friendly food choices, the more they feel that their parents and peers think it is important that they make these choices, and the more they experience that these choices can influence climate mitigation, the more likely they are to express intention to make climate-friendly food choices. Thus, our first hypothesis (H1) was supported.

**Table 1:** Correlations between the variables.

	1	2	3	4	5	6	7
1. Intention							
2. Attitudes	0.65***						
3. Subjective norms	0.30***	0.19***					
4. Outcome expectancy	0.42***	0.41***	0.30***				
5. Climate worry	0.49***	0.44***	0.26***	0.44***			
6. Climate optimism	0.08	0.06	0.04	0.09	−0.06		
7. Ambivalence	−0.34***	−0.34***	−0.04	−0.24***	−0.25***	−0.04	
8. Gender (0 = male;1 = female)	0.30***	0.26***	0.06	0.30***	0.37***	−0.13**	−0.22***

*** *p* < 0.001, ** *p* < 0.01, * *p* < 0.05.

Answering R2, which focuses on correlations in the extended TPB model, intention to make climate-friendly food choices was significantly negatively associated with objective ambivalence, and significantly positively associated with worry about climate change. This supports our H2, meaning that the more the young people harbor ambivalent attitudes about climate-friendly food choices, the less likely they are to have the intention to make these choices. Furthermore, stronger worry about climate change is linked to stronger intentions. As to the correlation between climate optimism and food-choice intentions, which we did not form a hypothesis about, it was positive but weak and statistically non-significant (*p* = 0.072).

As to the control variable gender, identifying as female (vs. identifying as male) correlated positively with all other variables except for subjective norms (*p* = 0.19; see Table 1).

### 5.2. Hierarchical regression analyses

We then answered R3, which focuses on the unique effects of the variables of the basic TPB model on the intention to choose climate-friendly food. After this, we answered R4 that asks if climate-related worry, optimism, and objective ambivalence explain unique variance in the intention to make climate-friendly food choice beyond the effect of the basic TPB model. In a hierarchical regression analysis (listwise deletion of cases with missing values), we first added the control variable gender (Model 1), and then added attitudes, subjective norms, and outcome expectancy (Model 2), and thereafter added climate-related worry, optimism, and objective ambivalence (Model 3). The results revealed that gender explains 9 percent of variance in the intention to make climate-friendly food choices, and the variables of the TPB model explain an additional 40 percent of variance (see Table 2). Attitudes, subjective norms, and outcome expectancy had unique statistically significant positive effects on behavioral intentions even when all these variables and gender were controlled for. These results support our third hypothesis (H3).

**Table 2:** Summary of a hierarchical regression model predicting intention to choose climate-friendly food.

	*R*^2^	*b (95% CI)*	*β*	*p*
Model 1	0.09			
Gender		1.08 (0.75, 1.40)	0.29	>0.001
Model 2	0.40			
Gender		0.44 (0.18, 0.70)	0.12	0.001
Attitudes		1.20 (1.04, 1.37)	0.55	>0.001
Subjective norms		0.26 (0.13, 0.40)	0.14	>0.001
Outcome efficacy		0.24 (0.09, 0.40)	0.12	0.002
Model 3	0.03			
Gender		0.31 (0.05, 0.58)	0.09	0.020
Attitudes		1.04 (0.86, 1.21)	0.47	>0.001
Subjective norms		0.24 (0.10, 0.37)	0.12	>0.001
Outcome efficacy		0.14 (−0.02, 0.29)	0.07	0.083
Climate worry		0.24 (0.13, 0.36)	0.16	>0.001
Climate optimism		0.13 (0.01, 0.25)	0.07	0.038
Ambivalence		−0.08 (−0.15, −0.02)	−0.09	0.017
*Adjusted R*^2^	*0.51*			
*N*	*449*			

As to the variables in the extended model (Model 3, see Table 2), we found them to increase the explained variance by 3 percent. The whole model explained 51 percent of variance in the intention to choose climate-friendly food. No concerns were detected regarding multicollinearity (Tolerance scores 0.68–0.96). Attitudes were still the strongest predictor of food-choice intention (*β* = 0.47, *p* < 0.001) but interestingly the effect of climate worry was stronger than those of the other variables of the basic TPB model (*β* = 0.16, *p* < 0.001). Subjective norms (*β* = 0.12, *p* < 0.001) and (female) gender (*β* = 0.09, *p* = 0.020) had positive unique effects, and objective attitudinal ambivalence had a negative unique effect (*β* = −0.09, *p* = 0.017), on behavioral intentions. However, the effect of outcome expectancy became non-significant (*β* = 0.07, *p* = 0.083). We studied this further and found that no single variable explained this, but that this effect became non-significant when all three variables of the extended model were simultaneously included. As to climate optimism, this effect became statistically significant in this regression model (*β* = 0.07, *p* = 0.038). Closer examinations showed that this is due to climate worry being added in the same model, which could indicate that worry suppresses some of the error variance in optimism, thus making the effect (albeit weak) significant. The results provide insights regarding the effects of objective ambivalence and optimism and support our fourth hypothesis (H4) regarding the unique effect of worry. However, the effect of optimism became statistically non-significant again in robustness tests, where we re-ran the analyses using either pairwise deletion (*r* = 0.06, *p* = 0.74) or replacing the missing values with the mean (*r* = 0.06, *p* = 0.69). No other results changed substantially in the robustness tests.

In answering the R5 we also examined each of the three interaction effects of interest in separate analyses. Here, our fifth and sixth hypotheses (H5, H6) were not supported, as the interactions between climate worry and optimism (*β* = −0.03, *p* = 0.417) and between climate worry and objective ambivalence (*β* = −0.01, *p* = 0.696) were non-significant, meaning that worry does not moderate the effect of optimism or objective ambivalence as we expected. The result, however, showed support for H7 as objective ambivalence interacted with attitudes (*β* = −0.08, *p* = 0.023) in having an effect on food-choice intentions (Δ*R*^2^ = 0.006, *p* = 0.023). We examined this interaction more closely by testing the correlation between attitude and behavioral intention in groups with low (below average) versus high (above average) ambivalence. The results showed that the correlation between attitudes and behavioral intention was stronger in the group with lower (*r* = 0.680, *p* < 0.001, *N* = 255) rather than higher (*r* = 0.515, *p* < 0.001, *N* = 206) ambivalence. The difference was statistically significant (*z* = 2.75, *p* = 0.006). The result indicates that, when experiencing attitudinal ambivalence, attitudes have a weaker influence on behavioral intentions compared to when no ambivalence was experienced.

## 6. Discussion

The aim of this paper was to examine what factors are connected to the intentions among late adolescents to make climate-friendly food choices. To do this, we examined the unique and combined effects of the variables of the TPB model (attitudes, subjective norms, and outcome expectancy), which we extended with emotional factors (climate-related worry and optimism) and objective attitudinal ambivalence.

The results supported the validity of the TPB model in explaining intentions to environmentally significant behavior (see, e.g., Yuriev et al., 2020), in this case making climate-friendly food choices among young people. Thus, it could be concluded that food-choice intention is associated with social and cognitive factors, meaning that the young people expressed higher intentions to choose climate-friendly food when they had positive attitudes toward this choice, perceived their parents and peers supporting it, and considered it to have an effect on climate-change mitigation. These are important results in a theoretical sense, because, although TPB is one of the most widely used models to explain behaviors and behavioral intentions (see, e.g., Kollmuss and Agyeman, 2002), thus far only few studies have investigated whether the model can be applied to explain climate-friendly food choices, and even fewer have studied the topic among young people. Thus, these results support the validity of using TPB also in this context, that is, concerning young people and climate-friendly food choices.

These results are also of importance from a practical perspective when the intention is to find ways to encourage young people to choose more climate-friendly food choices, considering that many of them are concerned about climate change and are searching for ways to engage with the issue (Hickman et al., 2021; Pihkala et al., 2022). Young people also have a huge collective impact on climate change in diverse ways, for example, through being consumers and citizens of today, influencers regarding parents and peers, and the future leaders of society. Food choices are among the most potent ways in which individuals can personally act on climate change, but this is still only weakly acknowledged in society (Wynes and Nicholas, 2017). For example, the influence of plant-based diet as a form of personal climate action is commonly underestimated, and people tend to resist diet changes more than the other possible forms of climate engagement (Macdiarmid et al., 2016; Wynes et al., 2020). Thus, it could be beneficial to provide more information about the role of food choices in climate-change mitigation, to inform the public about the important role that social support can play in promoting these choices, and to strengthen young people’s experience that their action can indeed make a difference.

We extended the TBP model by including factors that complement the specific rationality assumptions that are inherent in it. That is, this theory includes an assumption that behavioral decisions stem from rational calculations and conscious thinking processes, but we proposed that behavioral intentions can also be influenced by emotions. Thus, we added measures for two emotions: climate-change worry and optimism. In line with previous research showing the central role of climate worry in environmental engagement (Bouman et al., 2020; Ogunbode et al., 2022; Simon et al., 2022), we found that this emotion was the second strongest predictor of food-choice intention when the other variables were controlled for. This supports our suggestion that emotions, particularly worry, are important to consider in the climate context, and that they play an important role beyond the variables of the basic TPB model. This conclusion is strengthened by the observation that climate worry also correlated strongly with positive attitudes toward making climate-friendly food choices (*r* = 0.44) and with a sense of outcome expectancy (*r* = 0.44). Thus, while the causality cannot not be examined with our cross-sectional data, feelings of worry may direct individuals’ interest to the climate threat and motivate finding ways to help mitigate it, thereby increasing climate-friendly attitudes and the efforts of acquiring knowledge about how different kinds of behaviors can influence the climate problem at large. Even in societies that are not yet experiencing the dire consequences of climate change today, it seems rational to feel worried and to act on the situation, which is in many ways objectively serious. These results and interpretations are supported by the theory of affective intelligence claiming that worry/anxiety makes humans rational by making us use our entire capacity for higher-order thinking instead of relying on different kinds of heuristics (Marcus et al., 2000).

Interestingly, in the bivariate analyses, climate optimism did not correlate with food-choice intentions or with the variables of the basic or extended TPB model, and we did not find any interaction between optimism and worry in predicting intentions. Thus, the role of optimism in motivating or hindering environmental engagement is still unclear. This supports previous research results that have found no relation between climate optimism/hope and pro-environmental behavior (Hornsey and Fielding, 2016; Wang and Chen, 2022), but refutes other results (Feldman and Hart, 2018; Pihkala et al., 2022; Sangervo et al., 2022). However, it is important to point out that we did not measure a pure emotion of optimism, as was done in some previous studies (often in combination with other emotions such as hope) but rather a conception of certainty that climate change can be solved in the end. In addition, we did not focus on hope, which is different from optimism in that optimism is about certainty that things will turn out well in the end, while hope is related to uncertainty (Miceli and Castelfranchi, 2010). In some cognitive conceptualizations, hope consists of clear goals, pathways to reach these goals, and agency thinking (Snyder et al., 2001), which we did not focus on in this study. Therefore, in future studies it could be valuable to include either the emotion of hope (see Ojala, 2008) or some cognitive measure of climate hope (see Li and Monroe, 2019; Sangervo et al., 2022), or a more general trait or state measures of optimism (Kaida and Kaida, 2019; MacKinnon et al., 2022). It could also be that, just as is the case with the emotion of climate hope, one needs to consider what sources people base their optimism on, since they can, respectively, be more constructive or less constructive, seen from an engagement perspective (see Ojala, 2023). This would be important to study further also when considering that the effect of optimism became statistically significant (albeit very weak and non-robust) in our analyses when worry was added to the same model. It thus seems possible that these two variables interact, but not in the way than we hypothesized. For example, including worry in the analyses may suppress some error variance in optimism that could result from, for example, the different sources of optimism.

Also related to the specific assumptions of rationality in the TPB model, people do not necessarily have simply positive or negative views on the behavior. Thus, while they may generally have positive views regarding a certain behavior, the multiple—and sometimes conflicting—considerations regarding it can influence the decision to engage in it (for a review, see Conner and Sparks, 2002). Importantly, the bipolar scales that are commonly used in attitudes research fail to capture this complexity, because people may both agree and disagree with the statements (e.g., Breckler, 1994; Sparks et al., 2001). A related problem is that the midpoint of the Likert scales is often interpreted as reflecting a neutral stance but could also indicate uncertainty, indifference, or ambivalence (Breckler, 1994). Thus, we examined the role of ambivalence in explaining intention to make climate-friendly food choices. Previous research has found that a subjective sense of ambivalence influences climate-friendly food choices among young adults (Ojala and Anniko, 2020). Here, we studied whether similar results are found among late adolescents and strengthened the study by using a measure to capture ambivalence in a more objective way, thereby avoiding the problems with biased self-estimations regarding the presence of ambivalence (see Conner and Sparks, 2002). The index was built by first calculating the mean score for both positive and negative evaluations regarding climate-friendly food choices and then subtracting the difference between these evaluations from them. This approach has proven to be useful in previous research that has investigated the moderation effects of ambivalence across the TPB model (Povey et al., 2001; Sparks et al., 2001; Conner et al., 2003). Our results showed that objective ambivalence not only negatively influences the intentions to make climate-friendly food choices but also interacts with attitudes in predicting this outcome. Thus, when the young people have more ambivalent views on the climate-friendly food choices, they have a tendency to express fewer intentions to make them, and the effect of their attitudes on their intentions is weaker. More specifically, it is possible to both like and dislike making climate-friendly food choices, such as feeling these choices are important but simultaneously pointless, and these ambivalent views can decrease climate engagement. This is in line with previous research that has investigated how objective ambivalence may influence different behavioral intentions and outcomes (Povey et al., 2001; Sparks et al., 2001; Conner et al., 2003). These are important findings also in the light of the societal and collective aspects of climate action. Individuals may be ready to make a change, but such a change could be difficult to achieve if the hedonistic aspects, the need for a sense of empowerment, and the awareness of the importance of collective efforts are ignored. How people cope with this ambivalence could be valuable to explore in future research, as coping could either help them act despite their ambivalence or aggravate the ambivalence by the use of black-and-white thinking (see Ojala and Anniko, 2020; Ojala, 2022).

Yet another noteworthy finding is that while the bivariate correlation between outcome expectancy and intention to make climate-friendly food choices was relatively strong (*r* = 0.42), this correlation vanished when the variables of the extended model were controlled for in the regression analysis. Qualitative studies have shown that the conflicts behind the attitudinal ambivalence often concern outcome expectancy, in that people feel it is important to eat more climate-friendly/recycle more, but that they also feel that they are not able to really influence the larger environmental problems with their behavioral choices (see Ojala, 2008, 2022). Thus, it is perhaps not surprising that attitudinal ambivalence is one of the variables that could cancel out the effect of outcome expectancy when controlled for. Future studies could investigate further how the attitudinal factors, subjective norms, and emotions relate to the perception that food choices can have a positive influence on climate problems, and how these variables influence behavioral outcomes and intentions. It could also be valuable to replicate our results by including a measure for behavioral control, to test if this would yield more robust results. This could be important also when considering that many forms of climate action entail engaging in practices that young people have less control over, as they are not in charge of household practices if they still live with their parents.

There are also some limitations with this study that merit closer discussion. To begin with, we could not test the causal assumptions of the TPB using our cross-sectional data. It is thus difficult to draw conclusions regarding how the attitudes, perceptions of norms, and emotions develop and interact. We also could not measure changes in behavior and thus focused on behavioral intentions. This limits conclusions regarding how the variables included influence concrete outcomes. Cultural context also merits some consideration. The study was conducted in Sweden, which is a relatively wealthy and liberal Western country where young people tend both to be aware of the gravity of climate change and to have many possibilities of influencing their food choices (see Ojala, 2022). For example, Ojala (2022) has found that it tends to be easier for young people in Sweden to handle the potential disagreements regarding food choices with their parents than with their peers, which could reflect the democratic upbringing practices common in this country. Thus, future studies are needed across different cultural contexts, considering the culture-specific factors that may promote or hinder making climate-friendly food choices.

In conclusion, we have shown that the TPB model is useful in explaining intentions to make climate-friendly food choices among adolescents. Importantly, our results also show that people can have ambivalent views on these choices, which can weaken the effect of their attitudes on their behavioral intentions. Moreover, intentions to make climate-friendly food choices seem to reflect not only rational and conscious decisions but also climate-change worry. Thus, when studying and promoting climate-friendly food choices among young people, it is important to take into consideration both cognitive and emotional factors, as well as to acknowledge the complex nature of diet choices in our society.

## Data availability statement

The raw data supporting the conclusions of this article will be made available by the authors, without undue reservation.

## Ethics statement

The studies involving human participants were reviewed and approved by the Swedish National Ethics Committee (2019-03857). Written informed consent from the participants' legal guardian/next of kin was not required to participate in this study in accordance with the national legislation and the institutional requirements.

## Author contributions

MO contributed to the initial and developed conceptualization and design of the study, wrote some parts in the introduction, contributed to manuscript revision, organized the database, and was responsible for the collection of data. KJ performed the statistical analysis, contributed to the developed conceptualization, and wrote the larger part of the first draft of the manuscript. SO and AR contributed to the initial conceptualization of the study and manuscript revision. All authors contributed to the article and approved the submitted version.

## Funding

This research was supported by a grant to MO from the Swedish Research Council Formas [grant number 2017–00880] and a grant to KJ from the Swedish Research Council [grant number 2018–00782].

## Conflict of interest

The authors declare that the research was conducted in the absence of any commercial or financial relationships that could be construed as a potential conflict of interest.

## Publisher’s note

All claims expressed in this article are solely those of the authors and do not necessarily represent those of their affiliated organizations, or those of the publisher, the editors and the reviewers. Any product that may be evaluated in this article, or claim that may be made by its manufacturer, is not guaranteed or endorsed by the publisher.

## Footnotes

^1^Objective ambivalence = (P + N) / 2 – (∣P-N∣); (P = positive evaluation and N = negative evaluation; P as well as *N* were measured on unidimensional scales).
