# Supplementary material for: Climate-friendly food-choice intentions among emerging adults: extending the theory of planned behavior with objective ambivalence, climate-change worry and optimism
Source: Front Psychol. 2023 Jun 20;14:1178449. doi: 10.3389/fpsyg.2023.1178449 (PMC10319051; doi:10.3389/fpsyg.2023.1178449)
Supplement: Supplementary file 1 [file Presentation_1.pdf]

Supplementary Material

Climate-friendly food-choice intentions among emerging adults: Extending the Theory of Planned Behavior with objective ambivalence, climate-change worry and optimism

Front. Psychol. 14:1178449.doi: 10.3389/fpsyg.2023.1178449

Full scales used in the study

NB. The scales are translated from the original language, i.e., Swedish.

Intention to climate-friendly food choices

- Over the next 6 months, I will regularly make climate-friendly food choices.
- Over the next 5 years, I will regularly make climate-friendly food choices.

Scale: 1=Not at all likely; 7=Very likely

Attitudes

- My attitude towards making climate-friendly food choices is…

Scale 1: 1=Extremely negative; 3=Neutral; 5=Extremely positive

Scale 2: 1=Extremely unfavorable; 3=Neutral; 5=Extremely favorable

Outcome expectancy

- To what extent do you think that your food choices can affect the climate problem in a positive direction (reduce climate impact)?
- To what extent do you think that the Swedish people's choice of food can affect the climate problem in a positive direction (reduce climate impact)?

Scale: 1=Not at all; 2 = To a small extent; 3=To a rather small extent; 4=To a rather large extent; 5=To a very large extent

Subjective norms

- My mother thinks I should eat more climate-friendly food.
- My father thinks I should eat more climate-friendly food.
- My friends think I should eat more climate-friendly food.

Scale: 1=Not at all true; 2=Not entirely true; 3=True to some extent; 4=Quite true; 5=Absolutely true

Climate-change worry

To what extent are the following statements true for you?
- I worry that I will be negatively affected by the climate problem.
- I worry that my friends and/or my family will be negatively affected by the climate problem.

- I worry that future generations, i.e., people living in the future, will be negatively affected by the climate problem.
- I worry that animals and/or nature are or will be negatively affected by the climate problem.
- I worry that people living in poorer countries are or will be negatively affected by the climate problem.

Scale: 1=Not at all; 2 = To a small extent; 3= To a rather small extent; 4= To a rather large extent; 5= To a large extent; 6= To a very large extent

Climate-change optimism

Indicate how well the items apply to you:

- I believe that we will solve the climate problem in the future.
- I feel hopeful that we will fix the climate problem in the future.
- I think that the future looks relatively bright when it comes to our ability to deal with climate change.

Scale: 1=Does not apply at all; 2 = Does not apply that well; 3= Applies to a certain extent; 4= Applies fairly well; 5=Applies very well

Objective ambivalence

- Here are five positive judgements. When answering these: think only of positive feelings and thoughts and try to ignore any negative feelings and thoughts as we will ask about these later. Mark the extent to which you consider making climate-friendly food choices are…
  - Meaningful
  - Important
  - Pleasant to do
  - Well worth spending your time on
  - Associated with positive emotions

Scale: 1=”Not at all true” to 6=”Absolutely true”

- Here are five negative judgements. When answering these: think only of negative feelings and thoughts and try to ignore any positive feelings and thoughts that we asked about above. Mark the extent to which you consider making climate-friendly food choices are…
  - Meaningless
  - Unimportant
  - Unpleasant to do
  - A complete waste of time
  - Associated with negative emotions

Scale: 1=”Not at all true”; 6=”Absolutely true”
